# Association Between Hypernatremia and Delirium After Cardiac Surgery: A Nested Case-Control Study

**DOI:** 10.3389/fcvm.2022.828015

**Published:** 2022-03-08

**Authors:** Liang Hong, Xiao Shen, Qiankun Shi, Xiaochun Song, Lihai Chen, Wenxiu Chen, Shangyu Chen, Yingyin Xue, Cui Zhang, Jifang Zhou

**Affiliations:** ^1^Department of Intensive Care Unit, Nanjing First Hospital, Nanjing Medical University, Nanjing, China; ^2^Department of Anesthesia, Nanjing First Hospital, Nanjing Medical University, Nanjing, China; ^3^School of International Business, China Pharmaceutical University, Nanjing, China

**Keywords:** hypernatremia, delirium, cardiac surgery, risk factors, nested case-control

## Abstract

**Background:**

The association between hypernatremia and delirium after cardiac surgery has rarely been investigated. This study aimed to determine whether hypernatremia increases the risk of delirium after exposure.

**Materials and Methods:**

From April 2016 to June 2021, 7,831 consecutive patients receiving cardiac surgery were screened for potential enrollment. The primary outcome was postoperative delirium (POD). For the respective case of delirium, 10 controls were matched according to the index date within the nested case-control design. Hypernatremia exposure was defined as serum sodium > 145 mmol/L within 7 days before the index date. A generalized estimation equation was performed to assess excess risks for POD associated with hypernatremia, adjusted for demographics and clinical variables.

**Results:**

About 7,277 patients were included in the final analyses. About 669 (9.2%) patients with POD were assigned to the case group, and 6,690 controls were identified from the whole population. About 66.5% of the cases and 36.3% of the controls had hypernatremia exposure. After being adjusted to certain well-recognized confounding factors, hypernatremia showed a significant correlation with increased risk of delirium after cardiac surgery (adjusted OR, 1.73; 95% CI, 1.41~2.12). An e-value analysis suggested the robustness to unmeasured confounding.

**Conclusions:**

Hypernatremia was associated with an increased risk of delirium after cardiac surgery. This finding could have implications for risk stratification, early detection, and management of delirium in patients receiving cardiac surgery.

## Introduction

Delirium, marked by acute decline and fluctuation of attention, cognitive function, and disturbance of consciousness, refers to a common complication in patients receiving cardiac surgery. Delirium could result in poor prognosis, including prolonged intensive care unit (ICU) stay, longer duration of hospital stay, increased mortality risks, and long-term impairment of physiological function (1, 2). As revealed from existing studies, the incidence of delirium after cardiac surgery reached nearly 8~52% (1, 3–5). Unfortunately, the pathophysiologic cause of delirium remains unclear, and there has been no unique intervention or medication to treat delirium thus far. It is of great importance to recognize those patients at risk for or with delirium to immediately identify and remove the factors contributing to it (6–10). However, most well-recognized risk factors in delirium are non-modifiable, identification of modifiable risk factors is crucial for prevention and treatment of delirium after cardiac surgery.

Hypernatremia is a treatable biochemical disorder correlated with considerable morbidity and mortality. Patients receiving cardiac surgery are predisposed to developing hypernatremia as impacted by the administration of hypertonic solutions and renal-free water loss secondary to diuretics. Hypernatremia develops in 12–28% of patients receiving cardiac operation and contributes to 7–19% elevated risk of mortality (11–13). However, the impact of hypernatremia on delirium has not been fully explored yet. Rare studies focused on the relevant field (14–17), typically involving small sample sizes and drawing controversial conclusions. Accordingly, this nested case-control study was conducted to evaluate the association between exposure to hypernatremia and the risk of delirium after cardiac surgery.

## Materials and Methods

### Data Source

This retrospective study was conducted on patients admitted and receiving cardiac surgery at Nanjing First Hospital from April 2016 to June 2021. The data were collected from electronic medical record (EMR) databases. For EMR data collection and analysis, approval was gained from the Ethics Committee of Nanjing First Hospital (KY20170811-03).

### Study Populations

Patients satisfying the following criteria during the study period were recruited as the study objects: aged 18 years and older; received cardiac surgery, including but not limited to coronary artery bypass, heart valve surgery, aortic dissection (AD) repair surgery, etc. Exclusion criteria: Patients with preoperative cognitive decline and mental illness, such as dementia and intellectual disability; Patients with severe adverse events such as cardiac arrest during the operation; Patients with cerebrovascular accident (stroke) during the perioperative period; Patients who died or were discharged during the operation or within 7 days after the operation. Additionally, patients with incomplete information on exposure and outcome ascertainment, such as those without serum sodium measures or delirium assessment scale records, were excluded from the analytical risk set.

### Assessment of Delirium

Delirium was measured daily until discharge with confusion assessment method (CAM) (18) or CAM-ICU (19) for nonverbal (intubated) patients. The CAM diagnostic algorithm includes the following 4 characteristics: (1) acute onset of changes or fluctuations of mental status, (2) inattention, (3) disorganized thinking, and (4) an alerting level of consciousness. Patients who exhibited features of (1) and (2) and either (3) or (4) were defined as delirium. The CAM-ICU includes both brief cognitive testing and the CAM algorithm to determine the presence or absence of delirium. Assessment of delirium was performed by a nurse two times a day in ICU and one time a day in the general ward, confirmed by a doctor in charge.

### Hypernatremia Exposure

Hypernatremia exposure was defined as serum sodium >145 mmol/L within 7 days before the index date. More than one lab result, fulfilling the criteria of hypernatremia, was required to reduce the effect of experimental error.

### Study Design

We conducted a nested case-control study in the cohort to explore the association between hypernatremia and POD. We defined cases as patients who experienced delirium after cardiac surgery. We used the idea of counter-matching (20); 10 controls were randomly selected for each case from the entire cohort by the time of POD occurrence (index date) in this case. For each case, corresponding controls were at the risk of POD at the same index date at which the case occurred POD, no matter those controls had POD or not later.

### Confounding Variables

The data for confounders were derived from previous literature findings (1, 3, 4, 10, 21, 22) and hospital EMR dataset characteristics, and the following variables were included in the analyses: demographics: age, gender; comorbidities: hypertension, diabetes, myocardial infarction, hyperlipidemia, cerebral vascular disease, atrial fibrillation, chronic obstructive pulmonary disease (COPD), congestive heart failure, renal disease, liver disease; operation information: operation time, intraoperative blood pressure fluctuation, intraoperative blood loss, intraoperative blood transfusion, operation type; length of mechanical ventilation (MV) time, ICU stay and hospital stay before the index date. Renal disease was defined as a preoperative glomerular filtration rate <30 ml/min/1.73 m^2^ (body surface area) (23). Hyperlipidemia was defined as total cholesterol >200 mg/dL and/or triglyceridemic value >150 mg/dL. Other comorbidities were identified from a diagnosis before operation using the International Classification of Diseases, Tenth edition (ICD-10). The ICD-10 codes used for the identification of comorbidities are outlined in Supporting information (Table S1). Blood pressure fluctuation was calculated according to the formula: variance = $(\sum_{i=1}^{n}{\left(x_{i}-\bar{x}\right)}^{2})/n-1\;$, where *x*_*i*_ denotes a patient's mean blood artery pressure (MAP), and *n* represents the number of blood pressure measurements (24).

### Statistical Analysis

Measurement data conforming to a normal distribution were described as mean ± standard deviation. The student's *T*-test was employed for inter-group comparisons. Measurement data not conforming to normal distribution were denoted as median [lower quartile-upper quartile]. Wilcoxon rank-sum tests were performed to draw inter-group comparisons. The enumeration data were represented as frequency and percentage and compared by performing the Pearson χ2 test. Fisher's exact test was performed under the expected frequencies of one or more cells <5.

Since the patients in the control group were matched from the whole study population, all the patients with and without delirium may be selected as control when they were not diagnosed as delirium on the index date, and a unique patient could occupy as multiple observations on varied index dates, generalized estimating equation (GEE) was employed to estimate hypernatremia effects while accounting for correlation between multiple observations on the identical individual over time. The R version 3.6.3 was applied for statistical analyses. The values were considered with statistical significance at *p* < 0.05.

### Sensitivity Analyses

Several sensitivity analyses were conducted to evaluate the robustness of the effect of hypernatremia: (1) Alternative definition of hypernatremia exposure: hypernatremia in 3 days, 5 days, and 7 days before the event of interest; (2) Sodium and potassium were usually tested simultaneously from the same blood sample in the center of the authors. For this reason, hyperkalemia (serum potassium > 5.5 mmol/L) exposure was employed as the negative control to carry out another sensitivity analysis since no theory or study suggested the association between hyperkalemia and delirium; (3) We explored the potential for unmeasured confounding between hypernatremia and delirium after cardiac surgery by calculating E-values (25).

## Results

**Figure 1:**
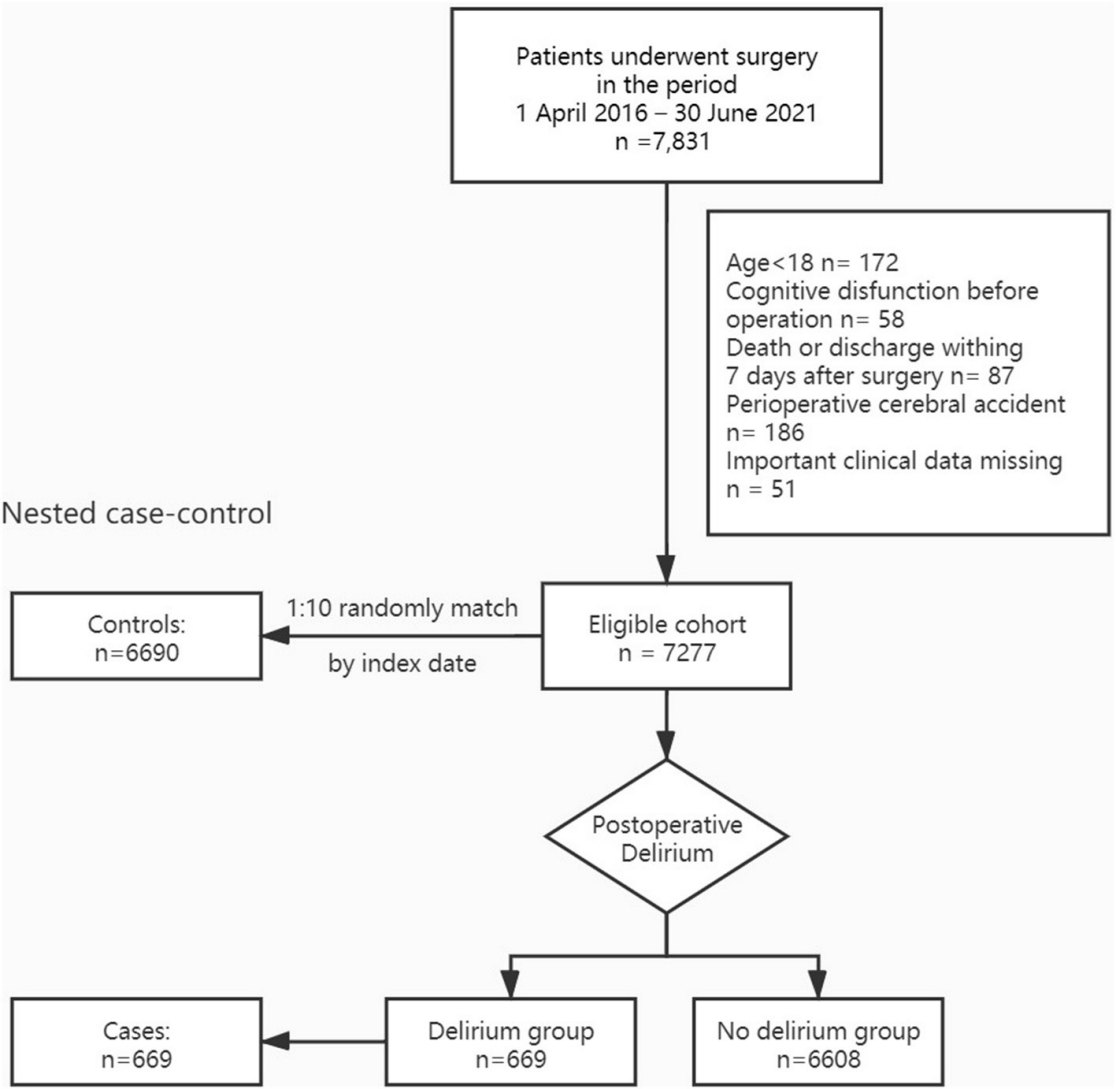
Flow chart of the study population.

Overall, the eligibility of 7,831 patients receiving cardiac surgery and admitted to the Cardiovascular ICU of Nanjing First Hospital, Nanjing Medical University, from April 2016 to June 2021 was assessed. Finally, 7,277 patients were included for analyses. Among them, 669 (9.2%) patients who occurred POD fell into the delirium group; other patients were no delirium group. All 669 patients in the delirium group were categorized in the case group. To improve the statistical efficiency, for the respective patient in the case group, 10 controls were randomly selected from the whole 7,277 patients still at risk for delirium at the same time after surgery as the case (Figure 1).

### Baseline Characteristics of the Patients in Delirium and No Delirium Groups

The incidence of POD in the population of this study was 9.2%. Table 1 presents the baseline characteristics of the patients with or without POD. The patients in the delirium group were older than those in the no-delirium group. The male patients were more dominant in the delirium group. The patients in the delirium group were more likely to have comorbidities of hypertension, cerebral vascular disease, kidney disease, and liver disease. The proportion of the patients with comorbidities of hyperlipidemia, atrial fibrillation, and heart failure in the delirium group was lower than those in the no-delirium group. Moreover, the operation time was significantly longer in the patients of the delirium group (5.54 ± 2.06 h vs. 4.12 ± 1.32 h, *p* < 0.001). Compared to patients without delirium, hemodynamic variability was larger among patients with delirium, with more blood loss and blood transfusion during operation. Also, more patients in the delirium group received coronary artery bypass (CABG) + Heart Valve Surgery and AD Repair Surgery when compared with the no-delirium group.

**Table 1:** Baseline characteristics of patients in delirium and no delirium groups.

**Variable**	**Delirium** **(***n*** = 669)**	**No delirium** **(***n*** = 6,608)**	***P*** **value**
Age (years)	62.64 ± 12.13	60.37 ± 11.72	<0.001
Sex (*n*, %)			<0.001
Male	488 (72.9 %)	3,835 (58.0 %)	
Female	181 (27.1 %)	2,773 (42.0 %)	
Comorbidities (*n*, %)			
Hypertension	450 (67.3 %)	3,212 (48.6 %)	<0.001
Diabetes	133 (19.9 %)	1,314 (19.9 %)	1
Myocardial infarction	47 (7.0 %)	376 (5.7 %)	0.187
Hyperlipidemia	95 (14.2 %)	1,447 (21.9 %)	<0.001
Cerebral vascular disease	91 (13.6 %)	638 (9.7 %)	0.002
Atrial fibrillation	105 (15.7 %)	1,556 (23.6 %)	<0.001
COPD	35 (5.2 %)	283 (4.3 %)	0.296
Heart failure	143 (21.4 %)	2,017 (30.5 %)	<0.001
Kidney disease	89 (13.3 %)	306 (4.6 %)	<0.001
Liver disease	50 (7.5 %)	240 (3.6 %)	<0.001
Operation time (h)	5.54 ± 2.06	4.12 ± 1.32	<0.001
Intraoperative MAP fluctuation	168.20 ± 92.28	176.31 ± 95.09	0.031
Intraoperative blood loss (ml)	1,200 [900, 1,550]	1,000 [800, 1,200]	<0.001
Intraoperative blood transfusion (ml)	410 [150, 900]	150 [0, 300]	<0.001
Type of surgery (*n*, %)			
Heart valve surgery	200 (29.9 %)	3,685 (52.7 %)	<0.001
Isolated CABG	155 (23.2 %)	1,821 (27.6 %)	0.017
CABG + Heart valve surgery	73 (10.9 %)	530 (8.0 %)	0.012
AD repair surgery (Open heart)	200 (29.9 %)	214 (3.2 %)	<0.001
Other procedures	41(6.1 %)	560 (8.5 %)	0.043

*COPD, chronic obstructive pulmonary disease; CABG, coronary artery bypass; MAP, mean artery pressure; AD, aortic dissection*.

### Baseline Characteristics of Patients in Case and Control Groups

Compared to controls, more patients in the case group had hypernatremia exposure (66.5 vs. 36.3%; *p* < 0.001). Besides, the patients in the case group were older and predominantly of the male sex when compared with those in the control. Compared to the patients in control, there were more patients with hypertension, cerebral vascular disease, kidney disease, and liver disease, and fewer patients with hyperlipidemia, atrial fibrillation, and heart failure in the case group. The operation time was markedly longer in the patients of the case group as compared to the control (5.54 ± 2.06 h vs. 4.25 ± 1.35 h; *p* < 0.001). Compared with the controls, the patients in the case group experienced more intraoperative blood loss and blood transfusion, and more patients in the case group received AD Repair Surgery. In addition, the patients in the case group had a longer duration of mechanical ventilation, longer time of ICU stay, and shorter time of hospital stay before the index date when compared to the control (Table 2).

**Table 2:** Baseline characteristics of patients in case and control groups.

**Variable**	**Case group** **(***n*** = 669)**	**Control group** **(***n*** = 6,690)**	***P*** **value**
Hypernatremia exposure	445 (66.5%)	2,431 (36.3%)	<0.001
Age (years)	62.64 ± 12.13	60.46 ± 11.86	<0.001
Sex (*n*, %)			<0.001
Male	488 (72.9 %)	3,917 (58.6 %)	
Female	181 (27.1 %)	2,773 (41.5 %)	
Comorbidities (*n*, %)			
Hypertension	450 (67.3 %)	3,284 (49.1 %)	<0.001
Diabetes	133 (19.9 %)	1,302 (19.5 %)	0.834
Myocardial infarction	47 (7.0 %)	369 (5.5 %)	0.127
Hyperlipidemia	95 (14.2 %)	1,537 (23.0 %)	<0.001
Cerebral vascular disease	91 (13.6 %)	667 (10.0 %)	0.004
Atrial fibrillation	105 (15.7 %)	1,626 (24.3 %)	<0.001
COPD	35 (5.2 %)	301 (4.5 %)	0.442
Heart failure	143 (21.4 %)	1,991 (29.8 %)	<0.001
Kidney disease	89 (13.3 %)	352 (5.3 %)	<0.001
Liver disease	50 (7.5 %)	219 (3.3 %)	<0.001
Operation time (h)	5.54 ± 2.06	4.25 ± 1.35	<0.001
Intraoperative MAP fluctuation	168.20 ± 92.28	174.50 ± 87.39	0.091
Intraoperative blood loss (ml)	1,200 [900, 1,550]	1,000 [800, 1,200]	<0.001
Intraoperative blood transfusion (ml)	410 [150, 900]	150 [0, 300]	<0.001
Type of surgery (*n*, %)			
Heart valve surgery	200 (29.9 %)	3,492 (52.2 %)	<0.001
Isolated CABG	155 (23.2 %)	1,849 (27.6 %)	0.015
CABG + Heart valve surgery	73 (10.9 %)	569 (8.5 %)	0.042
AD repair surgery (Open Heart)	200 (29.9 %)	281 (4.2 %)	<0.001
Other procedures	41 (6.1 %)	499 (7.5 %)	0.238
Length of MV (h)	20.17 [11.00, 41.50]	9.50 [7.00, 15.83]	<0.001
Length of ICU stay (days)	2 [1, 3]	1 [1, 2]	<0.001
Length of hospital stay (days)	7 [4, 12]	9 [7, 12]	<0.001

*COPD, chronic obstructive pulmonary disease; CABG, coronary artery bypass; MAP, mean artery pressure; AD, aortic dissection; MV, mechanical ventilation*.

### Association Between Hypernatremia Exposure and Delirium After Cardiac Surgery

We evaluated the association between hypernatremia exposure and POD by GEE analysis. The crude OR of POD was 3.48 (95% CI, 2.94~4.12; *p* < 0.001) for hypernatremia exposure; after being adjusted to all confounders, the OR of POD was 1.73 (95% CI, 1.41–2.12; *p* < 0.001) for hypernatremia exposure. Apart from hypernatremia, the GEE model adjusted for relevant confounders also showed that age, hypertension, kidney disease, liver disease, operation time, AD repair surgery, and length of mechanical ventilation were significantly associated with greater delirium risk; female sex and length of hospital stay were negatively associated with the occurrence of delirium events (Table 3).

**Table 3:** Association between hypernatremia exposure and delirium after cardiac surgery.

				**95%CI for OR**		
**Variable**	**Estimate**	**Wald**	**Adjusted OR**	**Lower**	**Upper**	***P*** **Value**
Hypernatremia	0.55	27.10	1.73	1.41	2.12	<0.001
Sex (Female)	−0.56	29.43	0.57	0.47	0.70	<0.001
Age (year)	0.03	51.75	1.03	1.03	1.04	<0.001
Hypertension	0.29	8.50	1.34	1.10	1.63	0.004
Diabetes	0.14	1.48	1.15	0.92	1.45	0.224
Myocardial infarction	0.23	1.62	1.26	0.88	1.80	0.204
Hyperlipidemia	−0.23	3.42	0.80	0.63	1.01	0.064
Cerebral vascular disease	0.13	0.80	1.14	0.86	1.52	0.37
Atrial fibrillation	−0.06	0.23	0.94	0.73	1.21	0.631
COPD	0.21	1.10	1.23	0.84	1.81	0.295
Heart failure	0.01	0.01	1.01	0.82	1.25	0.907
Kidney disease	0.37	4.93	1.44	1.04	1.99	0.026
Liver disease	0.70	13.03	2.02	1.38	2.96	<0.001
Operation time (h)	0.12	7.45	1.13	1.04	1.23	0.006
Heart valve surgery	−0.13	0.33	0.88	0.57	1.36	0.567
Isolated CABG	−0.17	0.66	0.84	0.56	1.27	0.416
CABG + Heart valve surgery	0.08	0.10	1.08	0.67	1.73	0.757
AD repair surgery (Open Heart)	0.94	13.96	2.57	1.57	4.21	<0.001
Intraoperative MAP fluctuation	0.03	0.12	1.03	0.86	1.24	0.729
Intraoperative blood loss (ml)	0.02	0.06	1.02	0.89	1.17	0.813
Intraoperative blood transfusion (ml)	0.10	3.24	1.10	0.99	1.23	0.072
Length of MV (h)	0.02	32.91	1.02	1.01	1.02	<0.001
Length of ICU stay (days)	0.05	2.18	1.05	0.98	1.13	0.14
Length of hospital stay (days)	−0.07	27.98	0.93	0.90	0.95	<0.001

*CI, confidence interval; OR, odds ratio; COPD, chronic obstructive pulmonary disease; CABG, coronary artery bypass; AD, aortic dissection; MV, mechanical ventilation; MAP, mean artery pressure. Generalized estimating equation analysis was performed using a logistic link function, clustered by unique patient id. Comorbidities were included as Boolean and type of surgery was included as dummy variables*.

**Figure 2:**
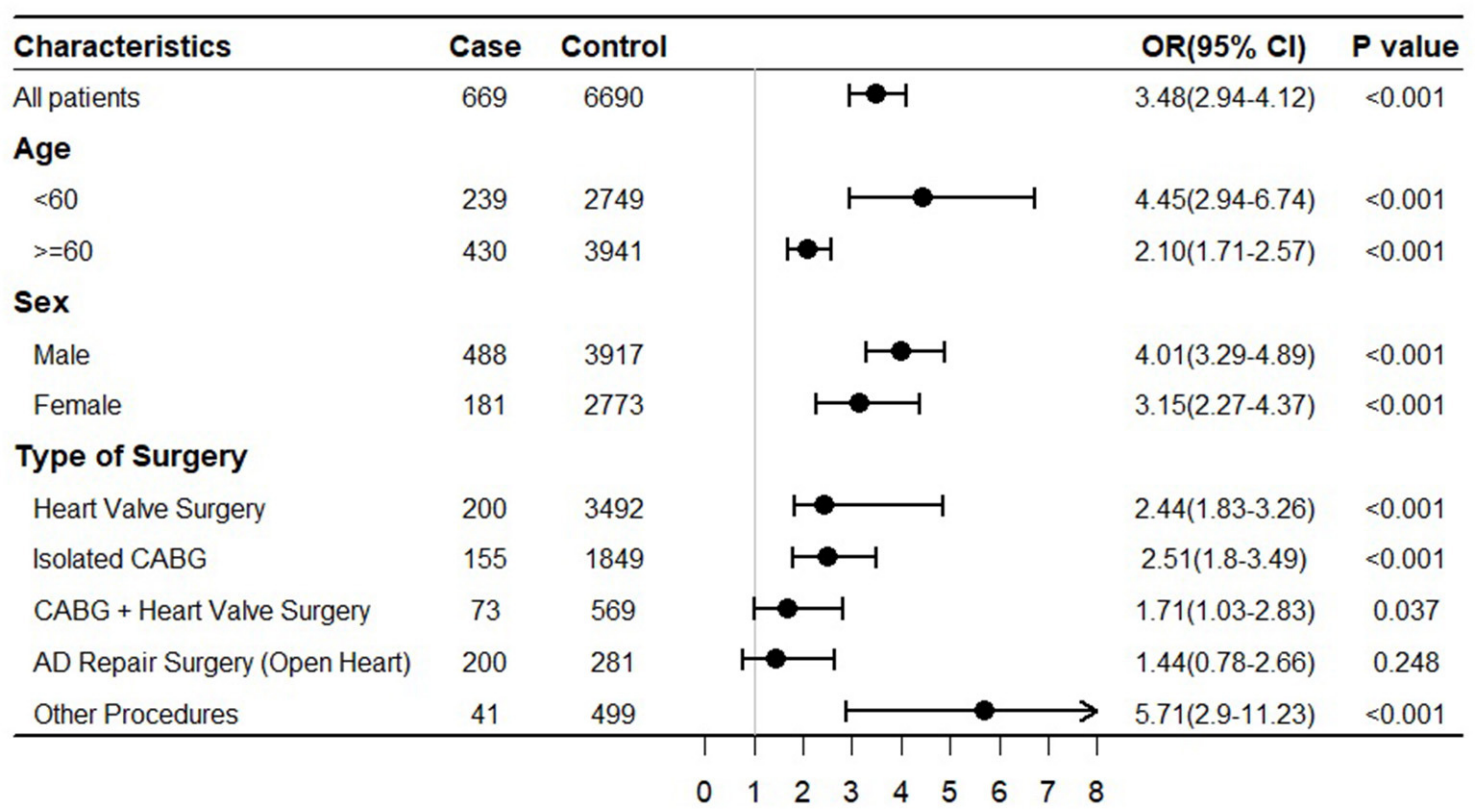
Subgroup analysis for delirium after cardiac surgery.

Subsequent subgroup analyses stratified by age, sex, and surgery type were performed (Figure 2). The results showed the consistent effect of hypernatremia on POD between subgroups. Crude ORs generated in each subgroup showed that the effect was stronger in male patients, younger patients (age < 60 years), and patients receiving other surgeries than AD repair surgery.

### Sensitivity Analysis

The sensitivity analysis showed a similar trend for association between hypernatremia and delirium. The relative risks appeared to be lower when the hypernatremia exposure assessment window is narrowed relative to the index date (Table 4).

**Table 4:** Sensitivity analysis for the duration of hypernatremia exposure on delirium.

**Variable**	**Crude OR**	***P*** **value**	**Adjusted OR**	***P*** **value**
Hypernatremia in past 3 days	3.27 (2.78~3.85)	<0.001	1.59 (1.30~1.95)	<0.001
Hypernatremia in past 5 days	3.39 (2.87~4.01)	<0.001	1.66 (1.36~2.04)	<0.001
Hypernatremia in past 7 days	3.48 (2.94~4.12)	<0.001	1.73 (1.41~2.12)	<0.001

*OR, odds ratio*.

Hyperkalemia exposure was adopted as a negative control in our analytical framework, and the presence of hyperkalemia was not associated with a POD (adjusted OR, 1.13; 95% CI, 0.86–1.48; *P* = 0.381).

Moreover, we generated an E-value to assess the sensitivity to unmeasured confounding. The E-value was 2.85 for the estimate and 2.17 for the lower confidence limit, which meant the finding was robust, unless an unmeasured confounder existed with a higher relative risk of delirium after cardiac surgery, with an OR higher than 2.85.

## Discussion

Delirium is a common complication in patients after cardiac surgery, leading to a longer duration of ICU stay and hospital stay. No effective intervention or treatment to treat delirium has been proposed, and risk assessment and reduction are likely to be the most effective approaches to managing delirium. In this nested case-control study, we generated a comparable sample of controls selected from the whole population as cases through matching, and hypernatremia exposure status was ascertained prior to the occurrence of delirium. Thus, the temporal relationship between hypernatremia and delirium could be reliably investigated. Finally, we found that hypernatremia exposure was associated with an increased risk of POD. After the association was adjusted to some well-recognized confounding factors, and the potential of unmeasured confounders was explored, hypernatremia was still significantly associated with delirium. Furthermore, it was a modifiable risk factor.

In the whole study population, 669 (9.2%) patients were diagnosed with POD. The incidence corresponded to reported literature but lay within the lower portion of reported ranges (1, 3–5). It is probably due to the difference of study population and exclusion of patients with cognitive dysfunction and a cerebral accident, which were recognized to be associated with increased risk of delirium (1, 3, 10). Tables 2, 3 showed that delirium was more likely to occur in elderly patients, male patients, patients with comorbidities of hypertension, cerebral vascular disease, kidney disease and liver disease, patients who received surgery with longer duration, surgery of AD repair, and patients who had longer MV and hospital stay time. The result indicated age, male, hypertension, cerebral vascular disease, kidney disease, liver disease, operation time, AD repair surgery were independent risk factors for delirium, which was consistent with previous studies (1, 3, 4, 10, 21, 22). In subgroup analysis, although all the point estimates of ORs were larger than 1, which suggested a consistent effect of hypernatremia on POD between subgroups, the 95% CI of OR spanned 1 in the AD repair surgery group. Possible reasons are as follows. First, AD repair surgery is performed under deep hypothermic circulatory arrest (DHCA), which could cause an extremely high incidence of both delirium and hypernatremia exposure (26), thus, making the odds of hypernatremia exposure among cases and controls less significant. Second, because of DHCA and other surgical trauma specific to AD repair surgery, the influence of surgery itself is predominant, weakening the effect of hypernatremia exposure.

The incidence of hypernatremia exposure in our patients was high (66.5% in the case group, 36.3% in the control group). On the one hand, we defined hypernatremia exposure as serum sodium > 145 mmol/L within 7 days before the index date rather than a continuous hypernatremia status in 7 days in this study. On the other hand, patients who underwent cardiac surgery are predisposed to develop hypernatremia. As shown in Table 2, 21.4% of the patients in the case group and 29.8% of the patients in the control group had heart failure before the surgery. The incidence of postoperative low cardiac output syndrome (LCOS) was not assessed in this study, and it could reach nearly 25% according to the previous study (27). Loop diuretics administration is reported as the primary therapy for perioperative fluid management to reduce the cardiac preload of those patients, which may induce hypernatremia. Elderly patients (age 60 years and above) accounted for 59% of our patients (Figure 2). In these patients, thirst impairment often occurred (28), and hypernatremia might be induced when thirst or access to water was impaired. Several preoperative conditions may predispose patients to deranged water balance; up to 20.4% of people with coronary artery disease or congestive heart failure have hypernatremia before surgery (13). Metabolic acidosis is the frequently encountered acute management challenge for patients receiving cardiac surgery; 30% of total cardiac cases have been associated with hyperlactatemia (29). Sodium bicarbonate is commonly used for the management of metabolic acidosis could lead to hypernatremia (26). In addition, factors such as intraoperative fluid management and cardiopulmonary bypass can cause hypernatremia as well (30–32).

Hypernatremia has been reported as an independent risk factor in delirium in previous studies (14–16). This study lends further support to this finding in a relatively larger, contemporary cohort of patients receiving cardiac surgery. The adoption of a nested case-control design could minimize the number of methodologic issues of the conventional case-control design, including selection bias and reverse causation. There are several considerations for the potential mechanisms. First of all, hypernatremia may affect the metabolism of the brain. Brain energy metabolism could be reduced with hypernatremia in animal experiments (33), and, as suggested from the latest study, impaired brain energy metabolism was causal for acute cognitive dysfunction (34). Furthermore, Na^+^ is critical to the uptake of amino acids. As reported by the study of Thurston et al. (35), hypernatremia could upregulate glutamic acid and γ-aminobutyric acid (GABA) levels in the brain in a dehydration model of mice. The change of GABA levels in the brain was recognized as one of the pathogenic mechanisms for delirium (36). Besides, cellular dehydration may be another potential reason since sodium contributes to tonicity and induces the movement of water across cell membranes as a functionally impermeable solute. Accordingly, hypernatremia invariably denotes hypertonic hyperosmolality, constantly causing cellular dehydration, at least transiently (37). Dehydration is a predisposing and precipitating factor in delirium (38). The dehydration could impair the physiological metabolic function of the cells, thereby causing organ damage including brain function, and thus, inducing delirium (39). Finally, hypernatremia may elevate plasma osmolality, thereby probably establishing an osmotic gradient from blood to the brain, thus, leading to net movement of water from the central nervous system and subsequent shrinkage in brain volume and further brain damage (33).

This study had several limitations. First, due to the inherent limitations of our data source, some risk factors like education and sleep deprivation could not be extracted from the EHR. We used E-value sensitivity analysis to quantify the potential implications of unmeasured confounders. Second, only the effect of hypernatremia exposure was analyzed without taking into account the duration and severity of hypernatremia since the time points of the serum sodium check were not predefined and well-organized. Lastly, this study was a single-center retrospective study, and more multicenter prospective randomized controlled trials are needed to further verify the results and explore potential causality.

## Conclusion

Hypernatremia was associated with an increased risk of delirium after cardiac surgery. This finding could have implications for risk stratification, early detection, and management of delirium in patients receiving cardiac surgery.

## Data Availability Statement

The original contributions presented in the study are included in the article/Supplementary Materials, further inquiries can be directed to the corresponding author.

## Ethics Statement

The study protocol was conducted in accordance with the Declaration of Helsinki and was approved by the Ethics Committee of Nanjing First Hospital, Nanjing Medical University (KY20170811-03). Informed consent was not obtained due to the observational and anonymous nature of data collection.

## Author Contributions

The concept of this study was conceived by LH, XSh, CZ, and JZ. Data were acquired by LH, XSh, QS, XSo, and LC. WC, SC, YX, and JZ participated in data analyses. LH prepared the first draft of the manuscript. All authors were involved in writing or editing the manuscript. CZ and JZ led the project and supervised the study. The manuscript is an original work, and the final version has been read and approved by all authors. All authors contributed to the article and approved the submitted version.

## Funding

This study was co-funded by the National Natural Science Foundation of China [81801891] and the Nanjing Medical Science and Technology Development Foundation [ZKX19021].

## Conflict of Interest

The authors declare that the research was conducted in the absence of any commercial or financial relationships that could be construed as a potential conflict of interest.

## Publisher's Note

All claims expressed in this article are solely those of the authors and do not necessarily represent those of their affiliated organizations, or those of the publisher, the editors and the reviewers. Any product that may be evaluated in this article, or claim that may be made by its manufacturer, is not guaranteed or endorsed by the publisher.

## Abbreviations

POD: postoperative delirium
COPD: chronic obstructive pulmonary disease
CABG: coronary artery bypass
ICU: intensive care unit
AD: aortic dissection
MV: mechanical ventilation
OR: odds ratio
CI: confidence interval
GEE: generalized estimating equation.
