# Supplementary material for: Association Between Hypernatremia and Delirium After Cardiac Surgery: A Nested Case-Control Study
Source: Front Cardiovasc Med. 2022 Mar 8;9:828015. doi: 10.3389/fcvm.2022.828015 (PMC8959150; doi:10.3389/fcvm.2022.828015)
Supplement: Supplementary file 1 [file Table_1.pdf]

Association between Hypernatremia and Delirium After Cardiac Surgery: A Nested Case-control Study

Supporting Information

Table S1 Codes used for identification of patients with comorbidities

**Comorbidities**	**ICD-10 code**
Hypertension	I10, I11, I12, I13, I15
Diabetes	E08, E09, E10, E11, E13
Myocardial infarction	I21, I22
Cerebral vascular disease	I60, I61, I62, I63, I65, I66, I67, I68, I69, G45, G46
Atrial fibrillation	I48
COPD	J40, J41, J42, J43, J44
Heart failure	I50
Liver disease	K70, K71, K72, K73, K74, K75, K76, K77
